# Defining our destiny: trainee working group consensus statement on the future of emergency surgery training in the United Kingdom

**DOI:** 10.1186/s13017-015-0019-4

**Published:** 2015-06-30

**Authors:** A. E. Sharrock, V. J. Gokani, R. L. Harries, L. Pearce, S. R. Smith, O. Ali, H. Chu, A. Dubois, H. Ferguson, G. Humm, M. Marsden, D. Nepogodiev, M. Venn, S. Singh, C. Swain, J. Kirkby-Bott

**Affiliations:** Association of Surgeons in Training (ASiT), Royal College of Surgeons England, 35 – 43 Lincoln’s Inn Fields, London, WC2A 3P3 UK; Department of Emergency Surgery, Southampton General Hospital, Tremona Road, Southampton, Hampshire, SO16 6YD UK

**Keywords:** Emergency general surgery, Surgical training, Surgical education, NHS National Health Service, Management and job planning

## Abstract

The United Kingdom National Health Service treats both elective and emergency patients and seeks to provide high quality care, free at the point of delivery. Equal numbers of emergency and elective general surgical procedures are performed, yet surgical training prioritisation and organisation of NHS institutions is predicated upon elective care. The increasing ratio of emergency general surgery consultant posts compared to traditional sub-specialities has yet to be addressed. How should the capability gap be bridged to equip motivated, skilled surgeons of the future to deliver a high standard of emergency surgical care? The aim was to address both training requirements for the acquisition of necessary emergency general surgery skills, and the formation of job plans for trainee and consultant posts to meet the current and future requirements of the NHS.

Twenty nine trainees and a consultant emergency general surgeon convened as a Working Group at The Association of Surgeons in Training Conference, 2015, to generate a united consensus statement to the training requirement and delivery of emergency general surgery provision by future general surgeons. Unscheduled general surgical care provision, emergency general surgery, trauma competence, training to meet NHS requirements, consultant job planning and future training challenges arose as key themes. Recommendations have been made from these themes in light of published evidence. Careful workforce planning, education, training and fellowship opportunities will provide well-trained enthusiastic individuals to meet public and societal need.

## Introduction

In the United Kingdom (UK), the National Health Service (NHS) seeks to provide high quality healthcare, funded by the taxpayer. Trainees are ‘invaluable eyes and ears in the hospital setting’ [1] and are well placed to evaluate day to day practice and areas for improvement. However, they remain an under-utilised resource in planning services. This Emergency General Surgery consensus document has utilised trainee perspectives of Emergency General Surgeons and the current provision of care for NHS acute surgical patients. Obstacles to a dedicated, high-quality future workforce, equipped to manage acutely unwell patients requiring surgical care have been identified, and strategies to overcome them have been recommended. The key overarching considerations at the Working Group meeting were; how can competency be reached and enthusiasm retained, and who is responsible for ensuring that each clinician maintains the high standard of skills expected by the UK population?

### The working group

A Working Group of 29 trainees ranging from Foundation Year (recently qualified) to the point of obtaining a certificate of completion of training (CCT), alongside a Consultant Emergency General Surgeon convened on 01 March 2015 during the Association of Surgeons in Training (ASiT) International Conference in Glasgow, UK. The aims of the Group were to address the training requirements for attaining the necessary emergency general surgery skills, and the formation of job plans for trainee and consultant posts. To achieve this, the Group was divided into four and all considered the following prompt questions:

- Will all newly appointed consultant general surgeons be emergency general surgeons?
- Can emergency surgical training be delivered through elective surgery?
- How should surgeons demonstrate emergency competence for completion of training?
- What should a job plan in emergency general surgery look like?
- What experience is necessary in trauma surgery, how should trainee surgeons obtain it, and how should this training be separate to emergency general surgery training?

The discussion was distilled with the full Working Group. Themes arising from the prompt questions are reported with concerns raised and relevant recommendations as bullet points.

To put these recommendations into context a thorough literature review of the clinical, service and training aspects of emergency surgical care provision was undertaken. The National Institute for Health and Care Excellence (NICE), Ovid (Medline and EMBASE) and PubMed libraries were searched to provide a clinical background. NHS England and the National Audit Office in conjunction with National Confidential and Public enquires were used to provide an insight of service provision. The Academy of Medical Royal Colleges, the four UK Royal Surgical Colleges, the Association of Surgeons of Great Britain and Ireland and the Intercollegiate Surgical Curriculum Project publications were reviewed for manning and training requirements. Searches were expanded through publication reference lists and related documents. This consensus statement seeks to identify the perceived ideal training and educational requirements of general surgical trainees to meet the current and future requirements of the NHS.

### Unscheduled general surgical care provision

In the UK the estimated population density is 413 people per square kilometre, compared to 35 and 3 people per square kilometre in the USA and some African nations respectively [2]. The relative density of the UK population means patients are often within a short distance of medical care, and that there is little requirement for the pan-specialty general surgeon. Instead, emergency orthopaedic, neurosurgical, cardiothoracic, ear nose and throat (ENT), maxillofacial, urological, vascular, and burns and plastics procedures are usually referred directly to the relevant speciality, and it is the remaining surgical conditions which are the responsibility of the general surgeon. This configuration is reflected by many European countries, who similarly have recognised the importance of providing training for emergency general surgeons, in an increasingly subspecialised environment [3]. Whilst the specifics of surgical provision vary between nations, the core need is to provide an emergency service with a well-defined range of skills.

Patients attending NHS hospitals with such emergency general surgical conditions make up half of all general surgery admissions in the UK [4]. Despite this, current models of UK healthcare tariffs and commissioning prioritise elective care over the care of emergency patients, through preferential financial agreements. A patient admitted as an emergency may not be physiologically prepared for a procedure, and particular consideration should be given to the growing demographic of the elderly patient, who may have multiple co-morbidities, cognitive impairment and frailty and whose mortality rate from emergency laparotomy currently sits at 24 % [5]. Equally concerning, is the wide variation in mortality for all-cause emergency laparotomy across UK trusts ranging from 3.6 – 41.7 %. This reflects the diverse range of models and standards used to deliver emergency surgical care [4, 5].

Some forward thinking institutions are developing and evolving their acute surgical care provision with dedicated specialist emergency general surgical clinical teams improving patient flow, treatments and outcomes. A recent single institution analysis of the hospital length of stay outcome of a new ‘acute surgical unit’ showed a 10 % reduction in bed days and cost saving of £475,848 per annum [6]. Whilst the NHS should evolve with evidence of best medical practice, it is a public institution, and therefore targets and restructuring are sensitive to manipulation by political agendas. It will take time to optimise the system. Going forward, consensus groups are a powerful tool to reflect on current systems and advise on the future direction of travel to improve patient care.

- Outcomes from emergency general surgery operations vary considerably across the UK
- The current system requires modification to improve outcomes and cost

### Emergency general surgery competency

Emergency surgery forms up to half of the general surgical workload in some specialities [4]. By its very nature it embodies an array of cross-specialty pathology, often in patients with multiple co-morbidities. Most of these admissions result from infective complications of disease processes. This is an area of pathology that has recently faded in significance, as political targets focus upon cancer care. We have now reached a point where a thorough re-evaluation of the management of surgical infection is required, as novel antibiotics developed in the second half of the twentieth century and new techniques gained through cancer surgery can be applied with a fresh approach to surgical infection in the twenty first century. Operations for such infective complications include laparoscopic cholecystectomies, laparoscopic or open appendicectomies and abscess drainages; they form the majority of procedures undertaken and are predictable in terms of training requirements. It is possible for transferrable skills in laparoscopic and open surgery and endoscopy to be instigated and developed in the elective setting, with other skills to be acquired during on-call shifts, providing staffing levels do not preclude training activities. Aside from these procedures and less complex intra-abdominal surgery, for example a perforated duodenal ulcer, there lacks a definition of what an emergency general surgeon should be capable of. Should the management of sepsis and surgical infection, as well as a more specialist gastrointestinal or trauma surgery skillset be an integral part of every emergency general surgeons’ abilities?

The minority of emergency cases include complex intra-abdominal pathology and trauma. Within this group it has been shown that most procedures (70 %) are performed for colorectal or small bowel pathology, whilst subspecialist oesphagogastric surgery, for example, comprises only 5 % of emergency laparotomies [7]. The emergency general surgeon is therefore required to know how to surgically intervene and perform a damage limitation procedure, with the ability to perform more sub-specialist surgery such as complex colonic or gastric resections if competent. It is entirely appropriate, and arguably expected, that a sub-specialist be contacted in complex cases, and that they may take the lead in the emergency context.

Every doctor who practises emergency surgery should be able to make timely, appropriate plans, and the surgical training pathway should provide the challenges and encouragement to equip each individual trainee with this skill. Consultant-delivered care is the accepted standard for high-quality expedient emergency surgery [8, 9], but this must be tempered with the opportunity for surgical trainees to safely accumulate the experience and skill necessary for future independent consultant practice.

- The exact competencies of the Emergency General Surgeon are yet to be defined
- Many emergency general surgery skills can be learnt in the elective setting.
- Management of sepsis and infective complications is an important aspect of care
- Consultant delivered care must be balanced with the need to train the next generation

### Trauma competence

The success of the British military trauma services in recent years [10], and a review of trauma services in 2007 [9, 11] sparked political interest in UK trauma management. The UK Major Trauma Centre (MTC) network was implemented in April 2012, and despite a significant proportion of injured patients now being treated in MTCs, a study of four large UK centres has shown that mortality, critical care requirements and length of stay are yet to significantly improve [12]. It is unknown what the role of limited UK trauma training will be on outcomes but it is clear that training in this area could be improved.

The technical skills required for competency in trauma surgery are far ranging and include surgical airway insertion, control of non-compressible haemorrhage through thoracotomy (left lateral and clam-shell), laparotomy and retroperitoneal access (pre-peritoneal packing and medial visceral rotation manoeuvres as examples), and control of contamination. The development of a list of trauma competencies was not within the remit of the Working Group, but questions were raised regarding the depth of training required to deliver a trauma service in the UK and how these skills are to be maintained. A focused trauma training curriculum should be developed and delivered in selected MTCs, and overseen with assessment criteria by the Intercollegiate Surgical Curriculum Project (ISCP) [13]. Future workforce planning can predict the number of trauma surgeons required at the MTCs; as only 22 of the 190 trusts providing an emergency surgery service also provide an adult major trauma service, only a proportion of emergency trained surgeons will need significant trauma training. It is not possible or necessary to train all emergency surgeons as trauma surgeons. Conversely, it may be possible that a flourishing trauma interest is developed in surgeons from other specialties, such as vascular surgery. Fellowships within high intensity trauma centres, or deployments to areas of operational theatres (in the case of military trainees), may be provided to bridge perceived knowledge gaps. Additionally, advanced trauma courses, simulation training and workshops need to be developed to augment the training available in the UK. All emergency general surgeons should be trained in the principles of surgical trauma care, and this could be delivered as a rotation to an MTC in higher surgical training.

- Trauma surgery and emergency surgery are distinct, and the definition of competence requires further work.
- Although many technical emergency surgical skills can be taught in the elective setting, this is not the case for trauma surgery proficiencies. Focused trauma surgical training curricula, to be delivered in highly selected units, should be developed.
- All emergency general surgeons should be aware of the principle of surgical trauma care, but not necessarily trained as trauma surgeons.

### Training to meet NHS requirements

The ratio of new to replacement consultant posts has increased [14], as have the number of ‘emergency general surgery’ consultant posts advertised since 2009 [15]. In response, there have been calls from some clinicians [16] for emergency general surgery to be a specialty in its own right. Following the implementation of the European Working Time Directive [17] it has been estimated that the exposure to surgical emergencies by the time of completion of higher surgical training is approximately 50 % less than those training 20 years ago [18]. There is currently no recognised higher surgical training programme for emergency general surgery, and consequently the emergency surgery and trauma competencies of those applying for emergency general surgery posts is largely unregulated.

Cognisant of the lack of consensus over the future of training for emergency general surgeons, the working group considered models in other countries. In Europe, there are a number of different models and the issues relating to operative exposure, training time and super-specialisation remain prevalent [3]. In the last few years the USA have trained Acute Care Surgeons who are competent in trauma surgery, surgical critical care and emergency general surgery. This has been partly driven by the profound decline in General Surgical Residency Applications and a shift in the desires of junior surgeons to have an acceptable work-life balance throughout their career. There was also recognition of the wide geographical variation in the provision of surgical trauma services and the need to address shortages [19, 20]. Consequently, the Acute Care Surgery training models are varied to suit local healthcare requirements whilst maintaining a minimum standard within the curriculum [21]. Geographical population density is less varied in the UK, so this flexible model of training less relevant.

Until now, the majority of emergency surgery training in the UK has been delivered yhrough time ‘on-call’. This model is no longer tenable in the reduced working hours models that have been instigated since the advent of the European Working Time Directive. Poorer patient outcomes for those admitted outside traditional working hours has created the current impetus for a seven day-working week for consultants and trainees [22]. Whilst it is difficult to separate service provision from training, the Working Group considered most on-call experience as service provision rather than an opportunity to obtain training proficiencies. This is beneficial to the organisation, and in some part to the individual, but must be balanced with the acquisition of clinical competency through training. Formal six-month rotations in emergency surgery with a targeted curriculum have provided some trainees with an immersion in the discipline. It is important that these placements are organised to complement the training of other trainees at the institution. This allows a sequential progression of skill acquisition rather than ‘ad hoc’ tuition. As Emergency units are set up and new knowledge is acquired, significant training opportunities for emergency surgery training in general surgery will present themselves. The decision-making and practical skills learnt in this context are transferable and add value to the trainees’ elective sub-specialty progression.

Prompted by a growing trend towards consultant-delivered care, trainees have found themselves supervised in the operating theatre more often than ever before. Undoubtedly, this is of patient benefit in the short term. Consultants need to pass on their knowledge to the next generation such that on completion of training, new consultants are not only capable of performing potentially complex procedures under pressure, but can also make informed, reproducible decisions throughout the operative process. This type of knowledge and processing is developed through repeated exposure to clinical challenges, and may not be refined in newly appointed consultants who have been sheltered from independent clinical practice. Surgeons in training need to be able to take the lead as well as being part of the team. The trainer-trainee relationship is key in providing the appropriate level of training for the individual. This raises the question as to whether all consultants are capable of training trainees to such a standard [23]. Uncoupling of the traditional ‘firm’ structure means that trainers may not be familiar with the competency of the trainee they are working with for a set day. This adds to the challenge of providing the appropriate level of training as trainers and trainees are increasingly unfamiliar with others experiences and expertise. Six month modular training in Emergency General Surgery may go some way to improving the working relationship between trainee and trainer and allow a structured progression of skills and experiences.

- The training pathway must be modified and monitored to meet the requirement to fill ‘Emergency General Surgery’ posts
- Formal placements in emergency general surgery may be suitable in structured surgical training programs.
- Training in non-technical skills, especially in the emergency setting, is essential.

### Emergency surgery consultant job planning

Emergency general surgeons may wish to have another sub-specialty interest that augments their emergency capability. This offers increased value to the population the surgeon serves and their employer. Importantly, some may experience a relative rest period from the intensity of continual emergency practice. Whilst possible, it is unlikely that sub-specialty interests such as Breast surgery and Vascular surgery (achieved via the new curriculum) will lend themselves to emergency general surgery due to their nature of elective practice, and specialty specific emergency workload in the case of vascular surgery. Some academic surgeons whose interests lie outside of the emergency field may also be unable to align themselves with emergency general surgery, equally others may pursue an academic interest in emergency surgery. Currently, as emergency general surgery posts are being established, there are almost as many different consultant job plans as posts [15]. Additionally, sub-specialty interest need not be limited to clinical sub-specialties: it may include academic, medical leadership, training and education fields. A survey of general surgical consultants and trainees showed that pure emergency general surgery job plans are not attractive to the current or future workforce [15]. There is a need for flexibility. Concerns have also been raised over the ability and desire to continue a high intensity high pressure work pattern in the later years of a surgeon’s career [24, 25]. To remain sustainable and attractive jobs, emergency general surgery roles will need to be flexible in terms of working hours (full-time or less than full-time), the combination of emergency and elective practice with variability over the course of a career, and welcome non-clinical sub-speciality interests.

- Job plans involving solely emergency general surgery are likely to be unattractive to the majority of surgeons and a sub-speciality interest (clinical or non-clinical) will need to be incorporated into job plans and the skillset maintained.
- Job plans will need to be flexible in terms of their component parts over the course of a career.
- It may be inappropriate to include emergency surgery in all consultants’ job plans: such as those undertaking significant academic or leadership roles.

### Future training challenges

The ‘Shape of Training’ review (‘ShoT’) [26] seeks to outline how doctors’ training pathways might be altered to meet a perceived change in patient needs. However, it is imperative that during this process, which risks shortening the length of training time further, quality and competence are not eroded. ShoT has recommended restructuring UK training in all specialties to form a broader base of practice, and has entertained the concept of credentialing to provide specialist qualification and capability later on. Whilst this remains contentious amongst some trainee groups, including ASiT, partly due to a lack of clarity over its implementation, it may represent an opportunity to redefine training in emergency surgery to meet an increasing service demand. However, no benefit can be gained from a training system producing unprepared sub-consultants requiring mandatory extra training before independent practise is truly possible. Whilst the Working Group does not agree that training should be shortened, if implemented it would mandate that focused, active, consultant-delivered training and reflective practice would be required alongside formal assessment through the Annual Review of Competence Progression (ARCP) process. Despite the difficulties it poses, this would most likely need to occur outside of prescribed service duties. The changes taking place in emergency general surgery provision may lend themselves to ShoT restructuring, but down-grading the quality assurance of a consultant by cutting corners and delivering a premature end product while maintaining the title does not benefit patients [27]. At best it would result in less competent consultants; at worst, accepted mediocrity and poorer quality healthcare. Patents rightly expect high standards from both consultants and trainees, and both require support to provide this level of care.

- The Working Group opposes the creation of a sub-consultant grade, but advocates quality training for independent consultant practice.

### Summary

This Working Group set out to address the training requirements for attaining the necessary emergency general surgery skills, and the formation of job plans for trainee and consultant posts. Through discussion and consensus, unscheduled general surgical care provision, emergency general surgery, trauma competence, training to meet NHS requirements, consultant job planning and future training challenges have been key themes. Recommendations have been based on evidence where possible and inferences drawn by group discussion. Trainees are well placed to consider how to bridge the training gap between their current state of competence and the ‘ideal’ breadth and depth of ability for an emergency surgeon. The training process for emergency general surgery and trauma should be tailored to service needs, but must be an attractive career option for ambitious and successful surgeons in training. Through combination of an interest in a related surgical discipline, workable job plan, and integration of training courses and a fellowship, well equipped highly capable emergency general surgeons will be available for work-force planning.

## Conclusions

The consultant workforce of the future must meet the patient demands for the NHS to work. Surgical trainees should be consulted regarding changes to surgical training; they are willing to engage in the process of optimising the training pathway. Careful workforce planning and design of education, training and fellowship opportunities, and flexible consultant job plans will enable the supply of well-trained individuals to meet demand, and retain enthusiasm. Above all, doctors who work within the NHS should not be overlooked to provide information on the functionality of a service, and forecast the direction of travel for future service improvement.

As a major stakeholder, ASiT is committed to pursuing high quality curricula, including that of emergency general surgery. ASiT opposes a sub-consultant grade or equivalent by another name, and does not agree that training should be shortened. Instead emergency general surgeons should be on the same contract and pay scale with the same opportunities for progression as their consultant colleagues in other sub-speciality areas.

## References

[1] Francis R. Report of the Mid Staffordshire NHS Foundation Trust Public Inquiry. http://webarchive.nationalarchives.gov.uk/20150407084003/http://www.midstaffspublicinquiry.com/report: 2013.

[2] Food and Agrigculture organisation and World Bank population estimates 2015, 1818 H Street, NW Washington, DC 20433 USA. http://data.worldbank.org/indicator/EN.POP.DNST

[3] Soreide K. Trauma and the acute care surgery model--should it embrace or replace general surgery? Scand J Trauma Resusc Emerg Med. 17. England 2009. p. 4. doi:10.1186/1757-7241-17-4. PubMed PMID: 19193218; PubMed Central PMCID: PMC2646681 10.1186/1757-7241-17-4 PMC2646681 19193218

[4] Royal College of Surgeons England Separating Emergency and Elective Surgical Care Working Party (2007). Separating Emergency and Elective Surgical Care: Recommendations for Practice.

[5] Saunders DI, Murray D, Pichel AC, Varley S, Peden CJ (2012). Variations in mortality after emergency laparotomy: the first report of the UK Emergency Laparotomy Network. Br J Anaesth.

[6] Pearson KCG, Trompetas V, Bailey I, Kirkby Bott J (2015). Cost benefit of setting up a dedicated acute surgery unit. Short Papers Br J Surg.

[7] Barrow E, Anderson ID, Varley S, Pichel AC, Peden CJ, Saunders DI (2013). Current UK practice in emergency laparotomy. Ann R Coll Surg Engl.

[8] Academy of Medical Royal Colleges. The benefits of consultant delivered care. http://www.aomrc.org.uk/doc_view/9450-the-benefits-of-consultant-delivered-care: 2012.

[9] National Confidential Enquiry into Patient Outcomes and Deaths. Trauma: Who cares? 2007.

[10] National Audit Office, Ministry of Defence (2010). Treating Injury and Illness arising on Military Operations.

[11] Darzi A. High Quality Care For All: Next Stage Review Final Report. http://webarchive.nationalarchives.gov.uk/20130107105354/http:/www.dh.gov.uk/prod_consum_dh/groups/dh_digitalassets/@dh/@en/documents/digitalasset/dh_085826.pdf. 2008

[12] Metcalfe D, Bouamra O, Parsons NR, Aletrari MO, Lecky FE, Costa ML (2014). Effect of regional trauma centralization on volume, injury severity and outcomes of injured patients admitted to trauma centres. Br J Surg.

[13] The Intercollegiate Surgical Curriculum Project, Educating the surgeons of the future. https://www.iscp.ac.uk/static/syllabus2013/gs_curric_2013.pdf.

[14] Kirkby-Bott J, Nelson M (2010). Trends in consultant posts advertised and SPRs completing training in general surgery. Bull Royal College Surgeons.

[15] Pearce L, Smith SR, Parkin E, Kennedy J, MacDonald A. Emergency General Surgery: emergence of a speciality by stealth (ASGBI abstract in print 2015). Association of Surgeons of Great Britain and Ireland conference abstract (ahead of print)

[16] Behar N, King D. Proposal for a new specialty: emergency general surgery. http://careers.bmj.com/careers/advice/view-article.html?id=20007882. 2012.

[17] Directive 2003/88/EC of the European Parliament and of the Council of 4 November 2003 concerning certain aspects of the organisation of working time. Official Journal of the European Union L 299, 18/11/2003. p. 0009–0019

[18] Association of Surgeons of Great Britain and Ireland. Issues in Professional Practice: Emergency General Surgery. Association of Surgeons of Great Britain and Northern Ireland. http://www.asgbi.org.uk/download.cfm?docid=A76F042A-0F5D-4D06-82BE71360E973708.

[19] Napolitano LM, Fulda GJ, Davis KA, Ashley DW, Friese R, Van Way CW (2010). Challenging issues in surgical critical care, trauma, and acute care surgery: a report from the critical care committee of the american association for the surgery of trauma. J Trauma.

[20] Green SM (2009). Trauma surgery: discipline in crisis. Ann Emerg Med.

[21] TraumaSource. The American Association for the Surgery of Trauma. Acute Care Surgery. http://www.aast.org/AcuteCareSurgery.aspx 10.1097/TA.0b013e3182569df4 22743396

[22] NHS England. NHS Services, Seven Days a Week Forum. Evidence base and clinical standards for the care and onward transfer of acute inpatients. http://www.england.nhs.uk/wp-content/uploads/2013/12/forum-summary-report.pdf.

[23] McIlhenny C, Pitts D. Standards for Surgical Trainers. Faculty of surgical trainers, Royal College of Surgeons of Edinburgh. http://fst.rcsed.ac.uk/media/9403/surgical-trainers-lr.pdf.

[24] Harkin D, Beard J, Wyatt M, Shearman C. Vascular Society UK Workforce Report. In: Society V, editor. http://www.vascularsociety.org.uk/wp-content/uploads/2014/07/VS-UK-Workforce-Report.pdf. p. p.25.

[25] Garrett K, Kaups KL (2014). The aging surgeon: when is it time to leave active practice?. Bull Am Coll Surg.

[26] Greenaway D. Securing the future of excellent patient care. The Shape of Training. http://www.shapeoftraining.co.uk/static/documents/content/Shape_of_training_FINAL_Report.pdf_53977887.pdf.

[27] Ferguson H, Fitzgerald E, Shalhoub J, Gokani V, Beamish A. The Shape of Training Review 2013: A Response on behalf of the Council of the Association of Surgeons in Training. http://www.asit.org/assets/documents/ShoT20Response20Final20Draft.pdf. 2013

